# Supplementary material for: In Situ Detection of Complex DNA Damage Using Microscopy: A Rough Road Ahead
Source: Cancers (Basel). 2020 Nov 6;12(11):3288. doi: 10.3390/cancers12113288 (PMC7694657; doi:10.3390/cancers12113288)
Supplement: Supplementary file 1 [file cancers-12-03288-s001.pdf]

cancers

MDPI

Supplementary Material

In Situ Detection of Complex DNA Damage Using Microscopy: A Rough Road Ahead

Zacharenia Nikitaki, Eloise Pariset, Damir Sudar, Sylvain V. Costes, and Alexandros G. Georgakilas

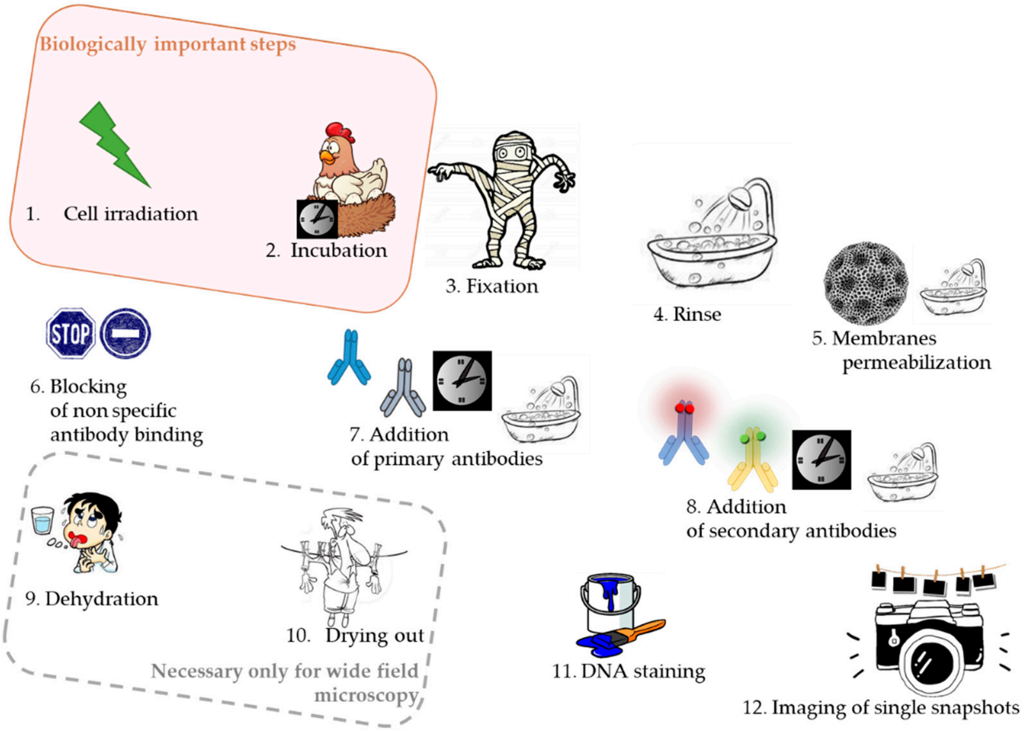

Figure S1. In situ immunofluorescence general protocol. Upon irradiation (1) the cells are let to rest and initiate to repair their DNA (2). When the specified time lapses, the cells are killed and fixated (3), thus a "snapshot" is created. Cells are rinsed (4) to remove fixation solution and their membranes undergo pemeabilization (5). This step is necessary for labeling antibodies to be able to penetrate into cell nuclei. Then, a mix of proteins (usually a serum) is added in order to prevent unspecific antibody binding (6). Incubation with primary (7) and secondary antibodies (diluted in blocking solution) follows. In the case of conventional wide field microscopy steps (9-10) are recommended. Suspension in alcoholic solution (9) and subsequent drying out (10) flatter the cells, making their nuclei to "fit" inside objective lens depth of field. DNA staining is necessary to make nuclei visible in fluorescence. Last step is the imaging (12). With this step the "biological snapshot" created at the 2nd step is stored as fluorescence image. We need to emphasize here that only the first two steps are of biological significance. The rest are technical details.

Table S1. Conceptual description of basic microscopy principles

Term	"Deconvolution"	Principle
**TEM**	Transmission electron M.	An electron beam transmits an ultrathin section of the specimen. The interaction between electrons and specimen creates the image.
**SEM**	Scanning electron M.	An electron beam scans the specimen surface. Secondary electrons emitted from the specimen are detected and they form the image.
**FRAP**	Fluorescence recovery after photo-bleaching	FRAP is a live cell monitoring technique, where the kinetics of proteins labeled by fluorescent probes is studied. A strong light source causes photo-bleaching of fluorescent signal, in the area of interest. Then the occurring diffusion towards the area of interest is monitored, given that other protein molecules will replace the photo-bleached ones.
**SPM**	Scanning probe M.	Scanning probe is a microscopy subcategory that uses a physical probe that scans the specimen surface.
**STM**	Scanning tunneling M.	STM is based on quantum tunneling effect. A conducting tip scans the surface, while a voltage difference is applied between the surface and the tip. Thus, electrons can tunnel the vacuum between tip and surface. The local density of states (solid state physics) is the main parameter to define the tunneling current. The variation of which, creates the image. Atomic level imaging.
**SNOM**	Scanning near field optical M.	Light in the near-field carries more high-frequency information and has its greatest amplitude in the region within the first few tens of nanometers of the specimen surface. Because the near-field light decays exponentially within a distance less than the wavelength of the light, it usually goes undetected. In effect, as the light propagates away from the surface into the far-field region, the highest-frequency spatial information is filtered out, and the well-known diffraction-based Abbe limit on resolution is imposed.
**AFM**	Atomic Force M.	A tiny cantilever scans specimen surface. While moving across the sample, due to its surface morphology, cantilever moves up and down. This transverse motion is recorded by a laser and its detector. The laser is directed to the cantilever head and its reflection there is recorded by the detector.

**2PEF**	2 Photon excitation fluorescence M.	Two photon (or later multi-photon) is confocal fluorescence M. where the fluorophore excitation is achieved by two (or more photons), that collectively have enough energy to excite the dye. Excitation beams travel from different directions to be met at the excitation site, causing thus the minimum photo-damage.
**PSTM**	Photon scanning tunneling M.	PSTM employs an optically conducting probe tip to map spatial variations in the evanescent and scattered field intensity distributions adjacent to a sample surface, which forms or is placed on the TIR surface.
**FCS**	Fluorescence correlation spectroscopy	FCS when employed in confocal microscopy, light is focused on a sample and the measured fluorescence intensity fluctuations (due to diffusion, physical or chemical reactions, aggregation, etc.) are analyzed using the temporal autocorrelation.
**PALM**	Photoactivated localization M	PALM super resolution M. is based on photoswitchable molecules. The resolved image is created gradually: A series of images of the same optical field is used. In each image different sparsely activated molecules are located. The resolved image presents the superposition of these located molecules.
**STORM**	Stochastic optical reconstruction M.	STORM super resolution M. is also based on photoswitchable molecules. The difference from PALM lies in the switching mechanism.
**3D SIM**	3D Structured illumination M.	SIM combines fluorescence, widefield-based structured illumination and digital image reconstruction. The structure, a sequence of known grating patterns, leads to the reconstruction of the image with up to two-fold improved resolution.
**SPIM or LSM (LSFM)**	Selective plane illumination M. Light sheet (fl.) M	While in conventional fluorescence the incident and fluorescent light are parallel and they both travel along the objective, in SPIM they are not. The fluorescent signal still travels through the objective to reach the detector, but the stimulation light in the form of a sheet is parallel to the objective focal plane.
**Bessel LSM**	Bessel light sheet M.	Bessel beams are non-diffractive nor spreading out. Bessel beams are also called “self-healing”, since after obstruction they are re-formed. Thereby, BB produce uniform and long enough light sheets, enhancing LSM resolving power.

**Lattice LSM**	Lattice light sheet M.	Optical lattices are periodic interference patterns in two or three dimensions created by the coherent superposition of a finite number of plane waves travelling in certain well-defined directions. Like an ideal Bessel beam, an ideal 2D lattice is non-diffracting in the sense that it propagates indefinitely in a direction y without changing its cross-sectional profile, which extends infinitely in x and z. In either case, this is accomplished by confining the illumination at the rear pupil plane of the excitation objective to points on an infinitesimally thin ring.
**STED nano-scopy**	Stimulated Emission Depletion	STED applies two laser pulses to localize fluorescence at each focal spot: The first laser pulse excites the fluorophore and the second depletes any fluorescent signals surrounding the excitation focal spot. The focal spot is raster-scanned across the sample to generate the high-resolution image.
**TRAM**	Translation M.	By TRAM a super-resolution image is restored from multiple diffraction-limited resolution observations using a conventional microscope whilst moving (translating) the sample in the image plane. TRAM can be implemented using any microscope, delivering up to 7-fold resolution improvement.
**SRRF**	Super-resolution Radial Fluctuations [1] [2]	SRRF is pretty similar to TRAM, whereas no sample moving is required. It can be adjusted to any microscope, it is suitable for live cell imaging and it also uses many frames from the same field of view. SRRF is a purely analytical super-resolution microscopy approach available as an open-source easy-to-use plugin for ImageJ. It does not require any photoswitchable fluorophores, although their usage increases SRRF's efficacy.

Table S2.: Ranking of common software and modules according to their citations in Google Scholar. LEFT Software used for colocalization and RIGHT ranking of the software alone.

rank	Software/ module AND coloc*	Google Scholar	NCBI	rank	Software/ module (alone)	Google Scholar	NCBI
	Colocalization*	1160 k	159 k				
1	ImageJ	83.2 k	34.3 k	1	ImageJ	376 k	191 k
2	FiJi	13.6 k	6.0 k	2	FiJi	125 k	20.8 k
3	Imaris	8.9 k	4.2 k	3	Imaris	28.4 k	11.9 k
4	JaCoP	3.6 k	1.7 k	4	CellProfiler	9.6 k	2.9 k
5	CellProfiler	1.8 k	703	5	JaCoP	4.2 k	1.4 k
6	CoLocalizer	263	85	6	CoLocalizer	256	88

Table S2. ranks some common freely available software and modules, according to their citations in Google Scholar, and their results in NCBI platform. In the screening two commercially available software, Imaris (maybe the most popular among close code) and CoLocalizer (a software dedicated to colocalization) were also included. In the left side of this Table, queries also include the colocalization filter, i.e. only publications that discuss colocalization, whilst in the right side ranking regards to the software alone. Moreover, in the first line of the table, results for the term colocalization are also presented. Queries for colocalization were: *[name of software/module] AND (colocalization OR colocalisation OR co-localization OR co-localisation).*

References

1. Gustafsson, N., S. Culley, G. Ashdown, D.M. Owen, P.M. Pereira and R. Henriques, *Fast live-cell conventional fluorophore nanoscopy with ImageJ through super-resolution radial fluctuations.* Nature Communications, 2016. **7**(1): p. 12471.
2. Culley, S., K.L. Tosheva, P. Matos Pereira and R. Henriques, *SRRF: Universal live-cell super-resolution microscopy.* The International Journal of Biochemistry & Cell Biology, 2018. **101**: p. 74-79.
